# Immune complexes from rheumatoid arthritis synovial fluid induce FcγRIIa dependent and rheumatoid factor correlated production of tumour necrosis factor-α by peripheral blood mononuclear cells

**DOI:** 10.1186/ar1926

**Published:** 2006-03-28

**Authors:** Linda Mathsson, Jon Lampa, Mohammed Mullazehi, Johan Rönnelid

**Affiliations:** 1 Unit of Clinical Immunology, Uppsala University, Uppsala, Sweden; 2 Unit of Rheumatology, Karolinska Institute, Stockholm, Sweden

## Abstract

Immune complexes (ICs) can induce production of cytokines by peripheral blood mononuclear cells via Fc receptors. Rheumatoid factor (RF) develop in response to ICs in many clinical and experimental settings. We investigated whether and how polyethylene glycol (PEG) precipitated ICs from rheumatoid arthritis (RA) sera and synovial fluid (SF) can influence cytokine production by peripheral blood mononuclear cells. We also examined the relationship between RF and IC induced cytokine production. Parallel sera and SF from 47 RA patients and sera from 15 healthy control individuals were PEG precipitated. The precipitates were added to serum-free peripheral blood mononuclear cell cultures and tumour necrosis factor (TNF)-α levels were measured after 20 hours. In separate cell culture experiments FcγRIIa and FcγRIII were blocked and monocytes were depleted or enriched. RF in serum was determined by nephelometry, and IgG levels in precipitates and anti-cyclic citrullinated peptide antibodies in serum were measured using ELISA. Clinical data were collected from the patients' charts. In two separate investigations, we demonstrated a correlation between RF, PEG-precipitated IgG levels and induction of the proinflammatory cytokine TNF-α by PEG-precipitated SF ICs. No such correlation was found for serum ICs. TNF-α levels induced by SF precipitates, but not serum precipitates, correlated with the number of swollen and tender joints. Monocytes/macrophages were shown to be the main responder cells, and blockade of FcγRIIa, but not blockade of FcγRIII, inhibited TNF-α production in cultures stimulated with precipitated ICs. Anti-cyclic citrullinated peptide correlated with RF but exhibited no association with IgG content in PEG precipitates or with precipitate-induced TNF-α levels. These findings support the hypothesis that SF ICs and correlated RF production are directly linked to cytokine-dependent inflammation in RA. Suppression of monocytes/macrophages in RA joints or blockade of the primate-specific activating FcγRIIa receptor might be ways to reduce IC-induced TNF-α production in the joints of seropositive RA patients.

## Introduction

Rheumatoid arthritis (RA) is a chronic inflammatory disease that mainly affects the joints. Rheumatoid factor (RF) is found in serum and synovial fluid (SF) of most RA patients [1], and the presence of RF is associated with a more aggressive and destructive disease course [2,3]. Although about 75% of RA patients are positive for RF, this state also occurs in other diseases and in healthy individuals in association with immune complexes (ICs) [1,4,5]. ICs can activate various cell types but a main target is the macrophage. Experimental IC-induced arthritis can be ameliorated by depletion of synovial macrophage-like cells before arthritis induction [6-8], suggesting that monocytes/macrophages play an important role in IC-induced joint inflammation. Moreover, IC stimulation of monocytes/macrophages [9] and monocytoid dendritic cells [10] has also been suggested to be of importance in RA pathogenesis [8,9].

ICs communicate with macrophages via Fcγ receptors, which results in phagocytosis, degranulation, transcription of cytokine genes and release of inflammatory mediators. Fcγ receptors have been shown to be important in the development of experimental arthritis. Several studies have shown that knockout mice that lack the activating FcγRIII are protected from IC-induced arthritis [11,12] whereas deletion of the inhibitory FcγRIIb induced arthritis in nonsusceptible mice [13]. There are important intraspecies differences in FcγR expression. The FcγRIIa receptor is expressed only in primates and not in rodents, and so can not be considered in FcγR studies in rodents. In humans, FcγRIIa has been proposed to function as the activating counterpart of FcγRIII [14], and is elevated in RA monocytes compared with those from healthy control individuals [14,15]. Blom and coworkers [9] demonstrated that FcγRII and Fcγ III expression was significantly higher on macrophages from RA patients compared with healthy control individuals, resulting in increased tumour necrosis factor (TNF)-α production following IC stimulation.

Recent therapeutic interventions such as anti-TNF-α and interleukin-1 inhibition show the importance of cytokines in RA [16]. Induction of proinflammatory cytokines via cross-linking of FcγR by ICs may be a possible mechanism of activation of cells in the rheumatic joint.

We previously reported that PEG precipitates known to contain high-molecular-weight ICs from systemic lupus erythematosus sera can induce interleukin-10 production from normal peripheral blood mononuclear cells (PBMC) via FcγRIIa [17]. Based on the hypothesis that RF production in RA mirrors IC production, we wished to investigate whether and how ICs from serum and SF of RA patients can induce cytokine production from mononuclear cells. We found an association between RF, IgG levels in SF ICs, and SF IC induced levels of TNF-α in RA; furthermore, the cytokine production was shown to be dependent on FcγRIIa on monocytes.

## Materials and methods

### Patients and healthy control individuals

We collected paired sera and SF from 47 RA patients (41 women and 6 men; mean age 55 years; age range 25–85 years) who fulfilled the American College of Rheumatology criteria for RA. The SF and serum samples were obtained in association with therapeutic arthrocenthesis. Clinical data were collected retrospectively from patient charts and included disease duration, C-reactive protein levels, erythrocyte sedimentation rate, number of swollen and tender joints, time lapse since preceding intra-articular steroid injection, and medications, including oral corticosteroids.

The patient samples were used in two investigations with partly different experimental set ups. In the first study sera and SF from 15 RA patients (13 women and 2 men; mean age 51 years; age range 25–85 years) and sera from 15 healthy control blood donors (six women and nine men; mean age 41 years; age range 25–65 years) were investigated. In the second study we focused on RA patients and investigated sera and SF from 32 RA patients (28 women and four men; mean age 57 years; age range 34–81 years). Out of the 47 investigated patients, 25 were treated with methotrexate, three with sulfasalazine, three with *Podophyllum emodi *glucosides (Reumacon^®^; Meda AB, Solna, Sweden), two with etanercept, one with auranofin, one with azathioprin, one with anakinra, one with a combination of sulfasalazine and methotrexate, and two with a combination of infliximal and methotrexate. Seven patients did not obtain any disease-modifying antirheumatic drug (DMARD) therapy, and DMARD data could not be obtained from one patient.

All patients and control individuals gave informed consent to participate in the study, which had been approved by the local ethical committees at the Karolinska University Hospital in Stockholm and the University Hospital in Uppsala.

### Polyethylene glycol precipitation of immune complexes

SF samples were incubated with 10 U/ml hyaluronidase (Sigma-Aldrich, Stockholm, Sweden) at 37°C for 30 minutes before polyethylene glycol (PEG) precipitation. Sera and hyaluronidase-treated SF were then mixed with equal volume of 5% PEG 6000 with 0.1 mol/l EDTA and left to stand at 4°C overnight before the precipitates were purified and washed in a single-step centrifugation procedure described previously [18]. Briefly, 1 ml phosphate-buffered saline (PBS) containing 5% human serum albumin (HSA) and 2.5% PEG 6000 (PBS-HSA-PEG) was added to 1.5 ml autoclaved Eppendorf tubes. Plastic cylinders made from 5 ml autoclaved pipette tips, by cutting off about 1.5 cm of the tips, were introduced into the Eppendorf tubes containing PBS-HSA-PEG. The SF or serum precipitated overnight were diluted 1:3 in RPMI-1640 containing 2.5% PEG 6000 and then placed on top of the PBS-HSA-PEG in the pipette tips. An interface was formed with the less dense, red RMPI-1640 solution on top. The tubes were then centrifuged at 2100 *g *and 4°C for 20 minutes; in this manner the precipitates in the upper 2.5% PEG-RPMI solution were centrifuged down to the bottom of the Eppendorf tube. The remaining PBS-HSA-PEG solution was removed and the pellet containing PEG-precipitated ICs was resolved in ice-cold sterile PBS to the original volume of SF or serum. The diluted PEG precipitates were placed on ice until their use in cell culture experiments.

### Preparation of peripheral blood mononuclear cells and cell cultures

Buffy coats obtained from healthy blood donors were diluted in PBS at room temperature and separated using a Ficoll-Paque Plus density gradient (Amersham Biosciences, Uppsala, Sweden). Following two washings in PBS, the cells were counted and diluted to 1 × 10^6 ^cells/ml in RPMI-1640 (Flow Laboratories, Irvine, Scotland, UK) supplemented with 1% glutamine, 1% penicillin streptomycin, 1% HEPES and 1% Ultroser G^® ^(Flow Laboratories). In previous studies conducted in our laboratory we found Ultroser G^® ^to sustain IC-induced cytokine production in otherwise serum-free systems (data not shown). Our experience of different responder cell populations used for IC stimulation show that PBMC populations may either be good responders to ICs or exhibit generally low or generally activated cytokine production without substantial effects of added ICs. Because of such variations, two PBMC donors were used as responder cells in parallel in each experiment. The results presented are from the PBMC donor giving the strongest net response on IC stimulation.

Freshly prepared PEG precipitates were added to the PBMC cultures (10% vol/vol) within two hours of preparation. Erroneous results were produced if PEG precipitates were frozen and thawed before cell culture experiments. Cells were then cultured for 20 hours in standardized 300 μl cultures before collection of supernatants. Initial experiments had shown this time point to be optimal for cytokine induction by ICs.

### Cytokine enzyme-linked immunosorbent assays

TNF-α levels were measured using two ELISA systems, namely whole antibodies in matched pairs (Cytoset CHC1754; Biosource Europe, Nivelles, Belgium) and F(ab')2 antibodies (Hu TNF-α Flexia CHC1751; Biosource Europe), following a recently described protocol [19]. Alkaline phosphatase was replaced by horseradish peroxidase (R&D Systems, Abingdon, UK) employing 3,3'-5,5'-tetramethylbenzidine (DAKO, Glostrup, Denmark) as substrate. Standard curves were constructed using recombinant TNF-α (R&D Systems).

In the first investigation we used the ELISA with whole antibodies. In the second study we found that 90% of the PEG precipitates contained detectable TNF-α levels (mean 47.01 pg/ml) using the whole antibody ELISA. Because this might have been an artefact caused by RF-like heterophilic antibodies in the precipitates, TNF-α analysis was repeated in the second study using F(ab')2 fragments of TNF-α antibodies and then 17% of the PEG precipitates were shown to contain low levels of TNF-α (mean 8.96 pg/ml). However, similar overall results were obtained in the second study with the ELISA using whole anti-TNF-α antibodies and F(ab')2-fragments of TNF-α antibodies. Subtraction of the TNF-α levels in the PEG precipitates from supernatant values using either ELISA did not change the general results presented below. Results are shown for TNF-α measurements using whole antibodies for the first study (*n *= 15 + 15) and F(ab')2 antibodies for both capture and detection in the second study (*n *= 32).

### Rheumatoid factor, IgG and anti-cyclic citrullinated peptide antibodies

RF levels in all serum samples were determined by nephelometry (IMMAGE Immunochemistry System; Beckman Coulter, Fullerton, CA, USA). The analysis was standardized using the international standard NIBSC 64/002 and the cutoff was set to 20 IU/ml. In a control group consisting of 100 healthy blood donors, two exhibited marginally positive values (20.4 and 21.6 IU/ml). We also tried to measure RF in SF but, probably as a result of the intrinsic light-dispersing properties of SF, we only obtained RF results from 59% of the RA SF samples using nephelometry, even following hyaluronidase treatment. The IgG ELISA used for measurement of IgG content in PEG precipitates was constructed not to be influenced by RF or heterophilic antibodies. As capture antibody we used a rabbit F(ab')2 directed against the IgG γ chain (A0407, diluted 1:640; DAKO). The detection antibody was a goat F(ab')2 antibody directed against the human IgG γ chain adsorbed against bovine immunoglobulins (109-056-098, dilution 1:10,000; Jackson ImmunoResearch Europe Ltd, Cambridge, UK). A well characterized normal human serum was used to construct a standard curve.

Serum anti-cyclic citrullinated peptide (CCP) was measured using the Immunoscan RA Mark II assay (Euro-Diagnostica AB, Malmö, Sweden) and the cutoff was set to 25 U/ml. In a control group consisting of 99 healthy individuals, two exhibited borderline positive reactivity (30 and 42 U/ml) and one exhibited high positive reactivity (1,643 U/ml).

### Monocyte depletion/enrichment

To investigate the hypothesis that monocytes were the main responder cells, monocyte enrichment or depletion antibody cocktails (RosetteSep™ StemCell Technologies, Vancouver, Canada) were added to heparinized blood and purification was performed in accordance with the manufacturer's instructions. This enrichment protocol yields totally untouched monocytes for subsequent functional studies. Depletion and enrichments were verified by staining with anti-CD14 FITC-conjugated antibodies followed by flow cytometric analysis. Cells depleted and enriched for monocytes were diluted in cell culture medium to the same total cell concentration and the same volume as used for untreated PBMCs, whereupon dissolved PEG precipitates were added to the cells.

### FcγR blocking experiments

Anti-FcγRII monoclonal antibody (IV.3 [Fab fragment]; Medarex, Nutley, NY, USA) or anti-FcγRIII (3G8 [F(ab')2 fragment]; Medarex) were added to the cells and left to stand at 4°C for 30 minutes before addition of dissolved PEG precipitates. The antibody concentration used was 1.5 μg/ml; preliminary experiments had shown equivalent blocking effect using either 1.5 or 4 μg/ml. Antibody IV.3 was previously shown to react specifically with FcγRIIa [20,21].

### Statistical analysis

To neutralize inappropriate impact of outliers, nonparametric statistics were used throughout the study. Mann Whitney *U *test was used for comparison between groups, Spearman's rank correlation test was used to evaluate correlations between quantitative variables, and Kruskal-Wallis test was used to investigate the association between DMARD therapies on the one hand and serum RF and *in vitro *TNF-α responses to PEG precipitates on the other. *P *<0.05 was considered statistically significant.

## Results

### Comparison of sera and synovial fluids from rheumatoid arthritis patients with healthy control sera

**Figure 1:**
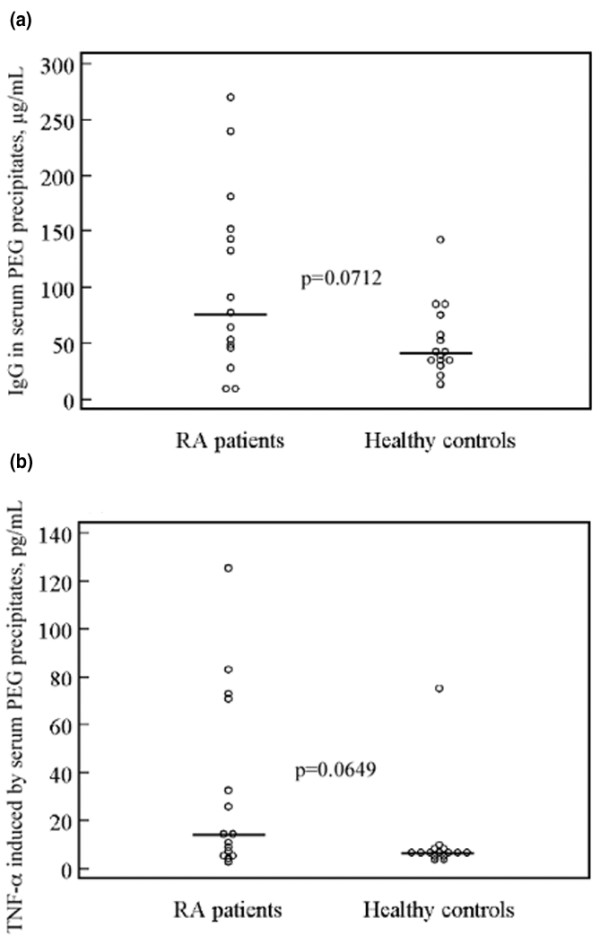
Trend for higher IC levels and IC-induced TNF-α levels in RA compared to control sera. Sera from 15 RA patients and 15 healthy control individuals were PEG precipitated and added to PBMC cultures and incubated for 20 hours at 37°C with 5% carbon dioxide, after which supernatants were harvested and TNF-α measured using ELISA. Nonsignificant trend toward **(a) **higher IgG levels and **(b) **greater TNF-α induction from RA precipitates as compared with healthy controls were apparent. Horizontal bars show the median value for each group. ELISA, enzyme-linked immunosorbent assay; PBMC, peripheral blood mononuclear cell; PEG, polyethylene glycol; RA, rheumatoid arthritis; TNF, tumour necrosis factor.

**Figure 2:**
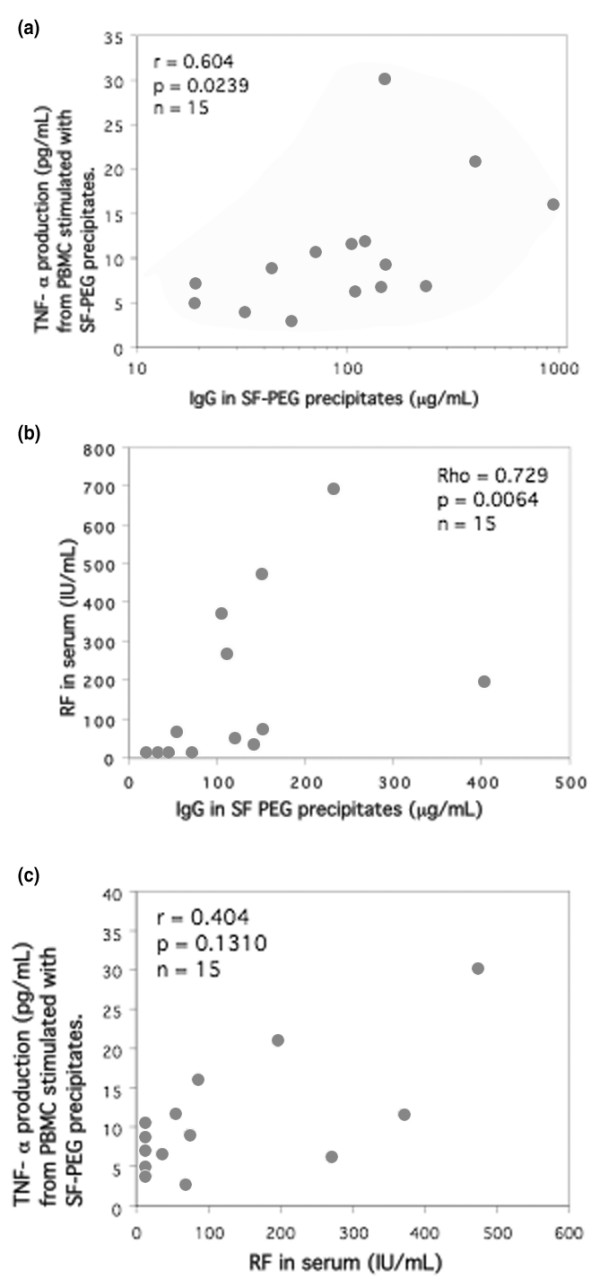
Correlation between SF precipitate induced TNF-α production, IgG levels in SF precipitates, and RF (*n *= 15). Healthy PBMCs were stimulated with PEG precipitates from SF from 15 patients with RA. The stimulated cells were cultured for 20 hours at 37°C with 5% carbon dioxide. IgG levels in the SF precipitates correlated with **(a) **TNF-α production after SF PEG stimulation and **(b) **RF measured in serum. **(c) **There was also a nonsignificant positive correlation between RF and TNF-α induced by SF PEG precipitates. Statistical analyses were performed with nonparametric tests to diminish the effect of outliers. PBMC, peripheral blood mononuclear cell; PEG, polyethylene glycol; RA, rheumatoid arthritis; RF, rheumatoid factor; SF, synovial fluid; TNF, tumour necrosis factor.

In the first study we compared paired sera and SF from 15 RA patients with sera from 15 healthy control individuals. We observed a nonsignificant trend toward higher IgG levels (*P *= 0.0712) and greater induction of TNF-α (*P *= 0.0649) by serum PEG precipitates from RA patients compared with healthy control individuals (Figure 1a, b). Although the levels of TNF-α induced by PEG precipitates from serum and parallel SF samples differed considerably in both directions in individual pairs, there was no statistically significant difference between TNF-α induction from RA serum or SF precipitates. We also found that IgG levels in the SF precipitates were significantly higher for RF-positive than for RF-negative RA patients (*P *= 0.0033; data not shown). A positive correlation was established between IgG levels in SF precipitates and TNF-α production from PBMCs stimulated with SF precipitates (*r *= 0.604, *P *= 0.0239; Figure 2a). There was also a strong positive correlation between IgG levels in the RA SF precipitates and RF measured in serum (*r *= 0.729, *P *= 0.0064; Figure 2b). We also found a link between TNF-α produced from PBMCs after stimulation with RA SF precipitates and RF measured in serum (Figure 2c). None of these correlations were evident for PEG precipitates obtained from parallel RA serum samples (Table 1).

### Correlation between tumour necrosis factor-α induction by synovial fluid precipitates, IgG content in the precipitates, and rheumatoid factor

**Figure 3:**
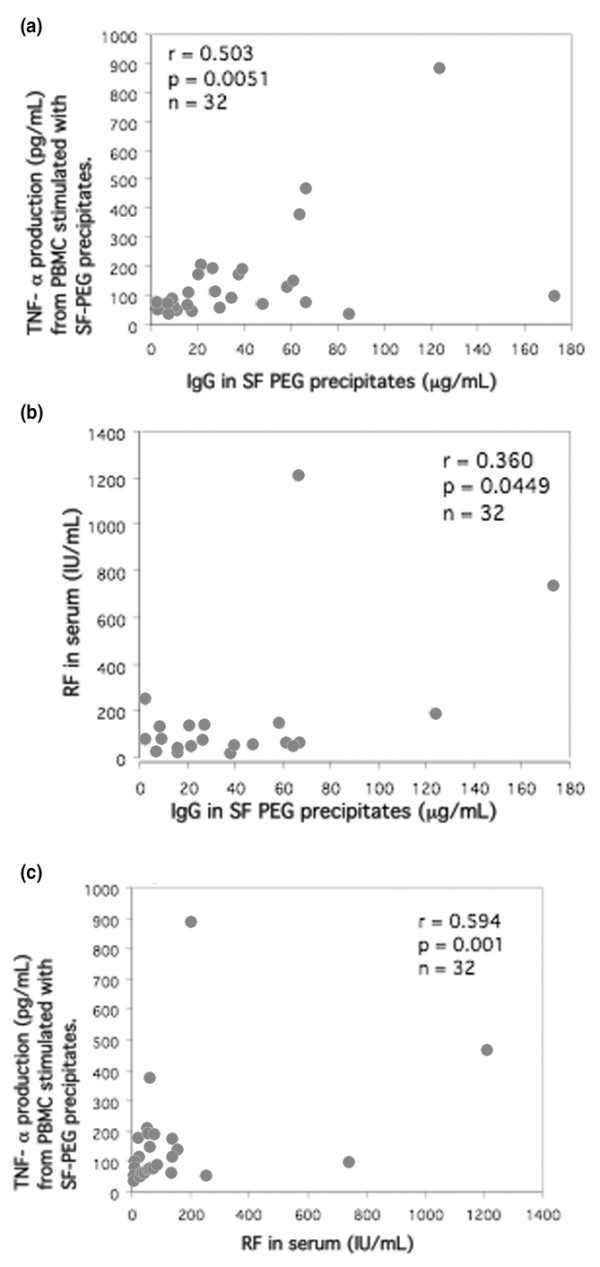
Correlation between SF precipitate induced TNF-α production, IgG levels in SF precipitates, and RF (*n *= 32). Healthy PBMCs were stimulated with PEG precipitates from SF from 32 patients with RA. The stimulated cells were cultured for 20 hours at 37°C with 5% carbon dioxide. IgG levels in the SF precipitates correlated with **(a) **TNF-α production after SF PEG stimulation and **(b) **with RF measured in serum. **(c) **There was also a correlation between RF and TNF-α induced by SF PEG precipitates. Statistical analyses were performed with nonparametric tests to diminish the effect of outliers. PBMC, peripheral blood mononuclear cell; PEG, polyethylene glycol; RA, rheumatoid arthritis; RF, rheumatoid factor; SF, synovial fluid; TNF, tumour necrosis factor.

In the second study with paired sera and SF from 32 RA patients we found the same association as in the first study between RF, TNF-α production following SF precipitate stimulation and IgG levels in SF precipitates (Figure 3, Table 1). On splitting the RA patients into RF-positive and RF-negative subgroups, the former exhibited significantly greater TNF-α production induced by SF precipitates (*P *= 0.0004; data not shown). We did not find any parallel correlations for the serum precipitates except for a weak correlation between RF and IgG content in the precipitates (*r *= 0.388, *P *= 0.0308; Table 1). We also tried to measure RF in SF but because of technical limitations we only got measurable RF values for 59% (19/32) of the samples. However, in these 19 samples there was a closer correlation between SF precipitate induced TNF-α and RF measured in SF (*r *= 0.667, *P *= 0.0047; data not shown) as compared with RF measured in serum (*r *= 0.284, *P *= 0.2279 [not significant]). Also, in this second study there was no significant difference in TNF-α levels induced by PEG precipitates from RA sera and SF.

### Anti-CCP levels correlate with rheumatoid factor but not with IgG content in PEG precipitates or with PEG precipitate-induced tumour necrosis factor-α levels

In the second study we measured levels of anti-CCP antibodies in serum samples, of which 26 out of 32 (81%) were anti-CCP positive. There was a positive correlation between anti-CCP and RF levels (*r *= 0.516, *P *= 0.0041; data not shown) in the serum samples, but we did not find any associations between anti-CCP and PEG precipitate-induced TNF-α production or IgG content in the precipitates (Table 1).

### Correlation between tumour necrosis factor-α production and number of swollen and tender joints

**Figure 4:**
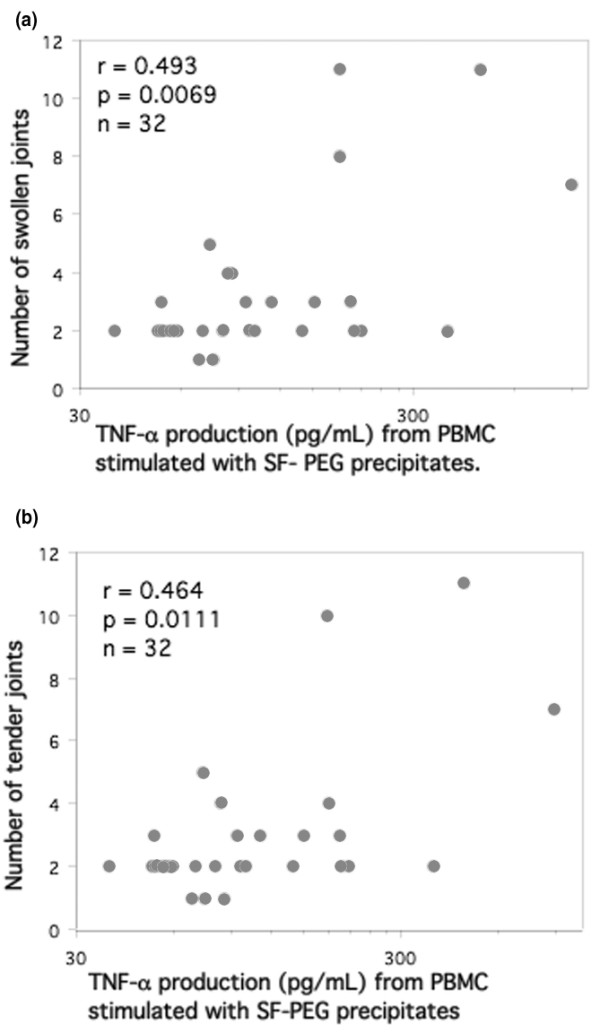
TNF-α production induced by SF precipitates correlate with the number of swollen and tender joints. PBMCs were stimulated with SF precipitates from 32 RA patients for 20 hours. TNF-α levels in the supernatants were measured using ELISA and data regarding the number of swollen and tender joints were collected from the patients' charts. The numbers of **(a) **swollen and **(b) **tender joints correlated with TNF-α production. Statistical analyses were performed with nonparametric tests to diminish the effect of outliers. ELISA, enzyme-linked immunosorbent assay; PBMC, peripheral blood mononuclear cell; RA, rheumatoid arthritis; SF, synovial fluid; TNF, tumour necrosis factor.

The amount of TNF-α produced after stimulation with SF precipitates correlated with the number of swollen and tender joints at the time of sampling (Figure 4a, b). No such correlation was found for stimulations with serum precipitates. We could not see any correlation between TNF-α production and age, sex, C-reactive protein, erythrocyte sedimentation rate, time since last intra-articular steroid injection, disease duration or medication, including peroral corticosteroids. There was no statistically significant difference in RF levels between patients treated with different DMARDs. However, all three patients treated with Reumacon^® ^and four out of seven of the patients not receiving any DMARD were RF negative.

### PEG precipitates from rheumatoid arthritis serum and synovial fluid induces tumour necrosis factor-α from monocytes

Paired sera and SF from two RA patients and serum from one healthy control individual were PEG precipitated and used to stimulate monocyte depleted, enriched, or unaffected PBMCs. TNF-α production was totally abolished when 99.9% of the monocytes were depleted. Conversely, when monocytes in the PBMC cultures were enriched from 7.5% to 54.7% monocytes, the TNF-α levels induced by serum precipitates were increased by between 15.5% and 27.4%. For the SF precipitates TNF-α production was increased to a greater extent (by between 45.4% and 63.1%; data not shown).

### Immune complex induced tumour necrosis factor-α production is partly mediated via FcγRIIa

**Figure 5:**
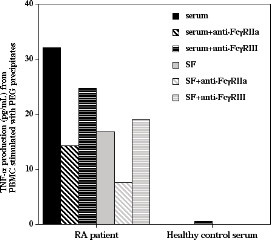
PEG precipitates from RA sera and SF induces TNF-α production via FcγRIIa.Anti-FcγRIIa and anti-FcγRIII antibodies were added to separate PBMC cultures before addition of PEG precipitates; culture was then continued for 20 hours. Anti-FcγRIIa antibodies blocked TNF-α production in cultures stimulated with PEG precipitates from RA sera and SF. One out of two experiments is shown. PBMC, peripheral blood mononuclear cell; PEG, polyethylene glycol; RA, rheumatoid arthritis; SF, synovial fluid; TNF, tumour necrosis factor.

FcγRIIa and FcγRIII were blocked to investigate receptor dependency of cytokine production induced by PEG precipitates. TNF-α production induced by the precipitates was reduced by 55% with blocking of FcγRIIa, but no effect of blocking FcγRIII was apparent (Figure 5). PEG precipitates from serum of healthy control individuals did not induce any or very low amounts of TNF-α and consequently did not exhibit any effect of FcγR blockade.

## Discussion

This, to our knowledge, is the first study to show an association between RF, IgG levels in SF ICs, and SF IC induced levels of TNF-α in RA. We also report that IC-induced TNF-α production is partly mediated via FcγRIIa with monocytes/macrophages as the main or perhaps only responder cells. These findings support the hypothesis that ICs in joints might provide a direct link to cytokine-dependent inflammation in RA, at least in RF-positive patients.

A stronger association between cytokine induction, IgG levels and RF was apparent for the SF precipitates than for serum precipitates, which is in agreement with the general belief that RF levels in serum reflect inflammation in the joints. RF has been associated with ICs in several diseases other than RA [1,5]. RF can also be produced after vaccination in healthy individuals during the time interval when antibodies and antigen form circulating ICs [4]. RF-producing B cells are present in the inflamed joints of RA patients [22] and RF measured in serum might therefore mirror the production of RF in RA joints. We also attempted to measure RF in SF, but for technical reasons we only achieved measurable values in 59% of the cases. Nonetheless, in the measurable subgroup of patients there was a considerably stronger association between SF PEG precipitate induced TNF-α production and RF in SF as compared with conventional RF measured in serum. This finding strengthens our hypothesis that serum RF is merely a reflection of RF produced in the inflamed joints in response to IgG-containing ICs with TNF-α-inducing properties. Moreover, our findings of stronger cytokine-inducing properties of ICs obtained from joints of RF-positive RA patients is consistent with the fact that seropositive RA is associated with a more severe disease outcome [2,3].

Anti-CCP antibodies have been shown to be highly specific for RA [23] and more strongly associated with joint destruction than RF [24]. As noted in several earlier studies, we saw a positive correlation between RF and anti-CCP in serum. However, we did not find any associations between anti-CCP and IC-induced TNF-α production or IgG levels in the PEG precipitates. Therefore RF *per se *and not the RF-correlated anti-CCP levels appear to be associated with IC-induced TNF-α and consequent joint inflammation.

PEG precipitation is a well recognized technique for the isolation of high-molecular-weight ICs. However, earlier investigations showed PEG-precipitated sera to contain uncomplexed immunoglobulins, C3 [25] and a number of serum proteins including fibronectin and albumin [26], besides IC containing IgG plus IgA and IgG plus C3. The view that PEG precipitates are composed only of ICs is therefore too simplistic. Because our cross-sectional approach employed a large number of ICs freshly prepared with an aseptic technique, we avoided the use of alternative, time-consuming techniques such as gel filtration and sucrose gradient centrifugation. To further determine IC content in our precipitates we measured IgG content in the precipitates and showed that IC-induced cytokine induction was dependent on binding to FcγRIIa that, because of its low affinity, preferably binds ICs over monomeric IgG [27].

Control experiments have shown that PEG precipitation of ultracentrifuged NHS or RF positive sera devoid of preformed ICs do not enhance TNF-α-inducing effects compared with serum added directly to the cell cultures without prior PEG precipitation. PEG precipitates from ultracentrifuged RF-negative NHS or from ultracentrifuged RF-positive sera induce comparable levels of TNF-α when they are added to responder PBMC cultures. These findings imply that neither PEG precipitation nor RF *per se *induce IC formation when no ICs are present initially. PEG precipitation on the other hand enhances TNF-α production when preformed ICs had been added to ultracentrifued NHS or RF-positive sera before PEG precipitation. An enhancing effect was also seen when nonaggregated IgG (Endobulin^®^; Baxter, Vienna, Austria) was added to ultracentrifuged RF-positive sera before PEG precipitation, probably because of minute amounts of dimer IgG in the preparation acting as small ICs.

In this cross-sectional study SF and serum samples were collected in association with therapeutic arthrocenthesis. Our finding that IC-induced TNF-α induction *in vitro *correlates with the number of swollen and tender joints at the time of sampling suggests that IC-induced cytokine levels might reflect a general quality of joint inflammation in individual patients. We are currently studying the cytokine inducing properties of paired SF samples from different joints obtained at the same time point, as well as paired SF samples from the same joint at different time points; in this way we aim to test the hypothesis that RF-associated induction of proinflammatory cytokines by joint ICs is a stable quality over space and time in individual patients with RA.

In the present study we examined the cytokine inducing effects of soluble ICs from RA SF. Collagen type II antibodies occur in a subpopulation of RA patients and these antibodies may form solid phase ICs at the cartilage surface in RA joints. We are currently investigating such ICs to obtain information regarding the similarities and dissimilarities between cytokine responses to soluble ICs (with hitherto unknown antibody specificities) obtained *in vivo *and artificial ICs created using well known autoantibodies directed against collagen type II [28].

Monocytes/macrophages were shown to be the main or perhaps only responder cells in the induction of TNF-α in our systems. The importance of monocytes in IC-driven joint inflammation is supported by earlier rodent experiments in which synovial macrophages were shown to play a central role in IC-induced arthritis models [6-8,29]. In addition, most disease-modifying drugs in RA are directed at suppressing monocytes and monocyte-derived cytokines [30]. Recent findings have also highlighted the importance of monocytes/macrophages [9] and monocyte-derived dendritic cells [10] in IC-induced cytokine production in RA joints.

Earlier studies conducted by Jarvis and coworkers [31,32] demonstrated cytokine-inducing properties of gel filtrated ICs from SF of patients with juvenile RA. Pretreatment of these ICs with native serum decreased subsequent cytokine production as compared with either pretreatment with heat-inactivated serum or no pretreatment [32]. These findings and our data on FcγRIIa-dependent cytokine production together argue that when ICs become heavily coated with complement, Fc fragments are covered by complement and prevented from interaction with Fc receptors, as was proposed by Nilsson [33].

Although complement activation by SF ICs is substantial, for two reasons we chose to study the effect of our ICs in a serum-free cell culture system. PEG precipitated ICs are known to carry covalently bound complement proteins after complement activation in the joint [25]. The amount of complement proteins on ICs from different joints therefore differs and is dependent on access to the classical complement cascade in the joints of individual patients. By exposing these ICs to a standardized native serum *in vitro*, all ICs will induce complement activation and differences between individual patients might diminish or disappear. When screening various cell culture systems we also found that a serum-free medium supplemented with Ultroser^® ^was superior to serum-containing cell culture media in sustaining IC-induced cytokine production. It was thereby also possible to investigate weak IC-induced responses that were not detected using other cell culture media formulations.

Although according to the literature RA SF may contain higher concentrations of ICs than RA serum [34], there was no significant difference between TNF-α levels induced by serum or SF precipitates. To be able to precipitate SF samples, hyaluronidase treatment was needed. Also, a number of joint-specific proteins such as partly degraded hyaluronic acid might be co-precipitated with SF ICs in parallel with what has been described for serum proteins [25,26]. Because of the experimental setup, we chose not to draw any conclusions from these findings of no difference, but instead we opted to concentrate on differences in cytokine responses between PEG precipitates from body fluids treated equally during the precipitation procedure.

Many studies have reported the importance of Fcγ receptors in the development of experimental arthritis. Thus, knockout mice lacking the activating FcγRIII have been shown to be protected from arthritis [13] and knockout mice lacking the inhibitory FcγRIIb develop arthritis on a nonarthritis susceptible background [13,35]. However, the effect of deleting FcγRIIb has not been consistent [36]. Rodents lack the primate-specific activating FcγRIIa, which has been shown to be elevated on RA monocytes compared with healthy control individuals [15]. Arguments are now accumulating that FcγRIIa might be a key activating mediator of IC-induced effects in humans and to act as the functional counterpart of FcγRIII in rodents [14].

In the present study, blocking of FcγRIIa resulted in markedly reduced IC-induced TNF-α production, indicating that the IC-induced cytokine production is at least partly mediated via FcγRIIa. We earlier reported that ICs from, for example, patients with systemic lupus erythematosus and artificial ICs can induce cytokine production via FcγRIIa, together with a correlation between IC-induced cytokine production and monocyte density of FcγRII, but not FcγRI or FcγRIII [17]. We also observed that ICs from patients with cryoglobulinaemia induces cytokine production via FcγRIIa [37]. Blom and coworkers [9] recently reported that the expression levels of FcγRII and Fcγ III are elevated on mature RA macrophages and that FcγR expression is correlated with IC induced levels of TNF-α [9]. Collectively, these data indicate an important role for FcγR expression on monocytes/macrophages in IC-induced inflammation in RA joints, and argue that FcγRIIa blockade is a possible means to suppress IC-driven inflammation in RA. However, a role for FcγRIII can not be excluded for two reasons. The anti-FcγRIII antibody 3G8 used in our studies has been shown occasionally to exert a nonspecific stimulatory effect on cytokine production [17,28]. Second, levels of monocyte expression of FcγRIII is low on unstimulated PBMC monocytes. In our earlier report [17] we investigated FcγR monocyte surface expression in 10 different PBMC populations. Whereas FcγRIII/CD16 exhibited low expression (median fluorescence intensity 39, as comparable with the nonspecific control antibody), levels of FcγRII/CD32 and FcγRI/CD64 were substantially higher (median fluorescence intensities 538 and 1133, respectively; data not shown). The selective importance of FcγRIII on inflammatory macrophages with increased FcγRIII surface expression [9] must therefore be investigated separately.

## Conclusion

We demonstrated a clear correlation between RF, IgG levels in PEG precipitated high-molecular-weight ICs from RA SF, and TNF-α production induced *in vitro *by these ICs. This supports the hypothesis that ICs are formed in the inflamed RA joint in parallel with RF production. Such ICs may then stimulate monocytes/macrophages in the joint to produce TNF-α via FcγRIIa stimulation. Suppression of monocytes/macrophages in the joints or blockade of the specific activating FcγRIIa receptor might therefore be a means to reduce IC-induced TNF-α production in the joints of seropositive RA patients.

## Abbreviations

CCP = cyclic citrullinated peptide; DMARD = disease-modifying antirheumatic drug; ELISA = enzyme-linked immunosorbent assay; HSA = human serum albumin; IC = immune complex; NHS = normal human serum; PBMC = peripheral blood mononuclear cell; PBS = phosphate-buffered saline; PEG = polyethylene glycol; RA = rheumatoid arthritis; RF = rheumatoid factor; SF = synovial fluid; TNF = tumour necrosis factor.

## Authors' contributions

LM planned the work, carried out the laboratory work and performed statistical analysis, as well as drafting the manuscript. JL collected the patient data and samples, and helped to draft the manuscript. MM helped with the laboratory work, and read and approved the final manuscript. JR participated in the design of the study, helped with the statistical analysis, and helped to draft the manuscript. All authors read and approved of the final manuscript.

## Figures and Tables

**Table 1:** Correlations between rheumatoid factor or anti-CCP and IgG content in immune complexes and TNF-α inducing properties of immune complexes

Correlation	Study 1 (*n *= 15)		Study 2 (*n *= 32)	
	
	*r*	*P*	*r*	*P*
RF/SF immune complexes				
IgG in SF precipitates/TNF-α induced by SF precipitates	0.604	0.0239	0.503	0.0051
RF/TNF-α induced by SF precipitates	0.404	0.1310 (NS)	0.594	0.001
RF/IgG in SF precipitates	0.729	0.0064	0.360	0.0449
RF/serum immune complexes				
IgG in serum precipitates/TNF-α induced by serum precipitates	0.171	NS	-0.265	NS
RF/TNF-α induced by serum precipitates	0.332	NS	0.329	NS
RF/IgG in serum precipitates	0.346	NS	0.388	0.0308
Anti-CCP/SF immune complexes				
Anti-CCP/TNF-α induced by SF precipitates	ND	ND	0.043	NS
Anti-CCP/IgG in SF precipitates	ND	ND	0.083	NS
Anti-CCP/serum immune complexes				
Anti-CCP/TNF-α induced by serum precipitates	ND	ND	0.191	NS
Anti-CCP/IgG in serum precipitates	ND	ND	0.246	NS

CCP, cyclic citrullinated peptide; ND, not done; NS, not significant; RA, rheumatoid arthritis; RF, rheumatoid factor; SF, synovial fluid; TNF, tumour necrosis factor.
